# Diminished State Space Theory of Human Aging

**DOI:** 10.1177/17456916231204811

**Published:** 2023-11-06

**Authors:** Ben Eppinger, Alexa Ruel, Florian Bolenz

**Affiliations:** 1 Institute of Psychology, University of Greifswald; 2 Department of Psychology, Concordia University; 3 PERFORM Centre, Concordia University; 4 Faculty of Psychology, Technische Universität Dresden; 5 Institute of Psychology, University of Hamburg; 6 Center for Adaptive Rationality, Max Planck Institute for Human Development, Berlin, Germany; 7 Science of Intelligence/Cluster of Excellence, Technical University of Berlin

**Keywords:** aging, cognition, decision-making, state space

## Abstract

Many new technologies, such as smartphones, computers, or public-access systems (like ticket-vending machines), are a challenge for older adults. One feature that these technologies have in common is that they involve underlying, partially observable, structures (*state spaces*) that determine the actions that are necessary to reach a certain goal (e.g., to move from one menu to another, to change a function, or to activate a new service). In this work we provide a theoretical, neurocomputational account to explain these behavioral difficulties in older adults. Based on recent findings from age-comparative computational- and cognitive-neuroscience studies, we propose that age-related impairments in complex goal-directed behavior result from an underlying deficit in the representation of state spaces of cognitive tasks. Furthermore, we suggest that these age-related deficits in adaptive decision-making are due to impoverished neural representations in the orbitofrontal cortex and hippocampus.

The use of new technologies—such as smartphones or public-access systems (like ticket-vending machines at train stations, or check-in counters at airports)—can be challenging for older adults (Czaja et al., 2006; Leung et al., 2012; Sengpiel, 2016). For example, many older adults prefer to buy train tickets at ticket counters rather than vending machines because they feel unable to navigate the complex multioption displays of these devices (Schreder et al., 2012; Sengpiel, 2016). Thus, a technology that is meant to provide faster and more convenient access to public transport may turn out to be a barrier for populations such as older adults. What this example means to illustrate is that many new technologies require learning and representational abilities that, with healthy aging, become more limited. This may turn out to be a problem for Westernized societies that (a) rely more and more on these technologies (Malik, 2014) and (b) have a growing proportion of older adults (United Nations, 2019).

In this article, we provide a cognitive (neuro)scientific perspective on the potential psychological and neurocomputational underpinnings of these age-related impairments in complex learning and decision-making. The main theoretical argument that we develop in this work is that the difficulties in adaptive behavior seen in older adults may arise from an underlying deficit in their ability to learn and represent state spaces of cognitive tasks (see Fig. 1). In line with previous work (Wilson et al., 2014), we define the *state space* of a task as an abstract representation of the structure of the task—that is, the states that it involves and the available actions and the transitions between states that may follow from these actions (note that this definition of a state space is broader than in other parts of the literature; e.g., Liu et al., 2021; Sutton & Barto, 1998). In terms of the example provided in the introduction, the state space of a ticket-vending machine consists of a set of menus (states) that contain choice options and the corresponding actions that link the different states (transitions to the next menu). To buy a ticket, the user (agent) must navigate and make sequential decisions in this state space. In many instances, the state space may not be directly observable and must therefore be learned from experience. In terms of the underlying neurobiological mechanisms, we suggest that functional decline in the representational capacity of the orbitofrontal cortex (OFC) and the hippocampus (HC) are the major source of the observed behavioral deficits (Bornstein & Daw, 2013; Bradfield et al., 2015; Kaplan et al., 2017; Schoenbaum et al., 2009; Wilson et al., 2014).

**Fig. 1.**
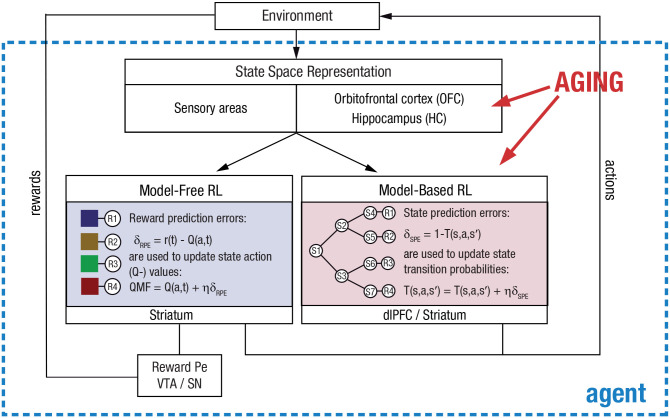
Diminished state space theory of human aging. This theory suggests that age-related impairments in goal-directed (model-based) reinforcement learning (RL) and decision-making result from a diminished representation of state spaces of tasks in the hippocampus and orbitofrontal cortex (OFC). In sequential decision tasks, older adults primarily rely on model-free (MF) learning, which has been associated with dopaminergic reward prediction-error (Pe) input from the ventral tegmental area (VTA) and substantia nigra (SN) to the striatum. In contrast to younger adults, they show deficits in model-based (MB) learning which has been suggested to depend on state prediction-error signals from the dorsolateral prefrontal cortex as well as reward information from the striatum. dlPFC = dorsolateral prefrontal cortex; RPE = reward prediction errors; SPE = state prediction errors.

## Empirical Evidence

### Age-related impairments in model-based learning result from deficits in the representation of state spaces

Recent computational work on reinforcement learning in humans has focused on two different decision strategies: model-free decision-making, which relies on the learning of simple associations between states or actions and reward, and model-based decision-making, which relies on the learning of a state space (model) of the environment that is used for forward planning of actions (Daw et al., 2011; Gläscher et al., 2010). The advantage of model-free decision-making is its computational efficiency and low cost in terms of cognitive resources. The downside of this strategy is that it is inflexible and slow to adapt to changes in the environment because the agent has to relearn the contingencies from experience. In contrast, the advantage of a model-based strategy is its flexibility: If rewards or contingencies between states in the environment change, the learner can quickly adapt behavior by updating the state space representation.

**Fig. 2.**
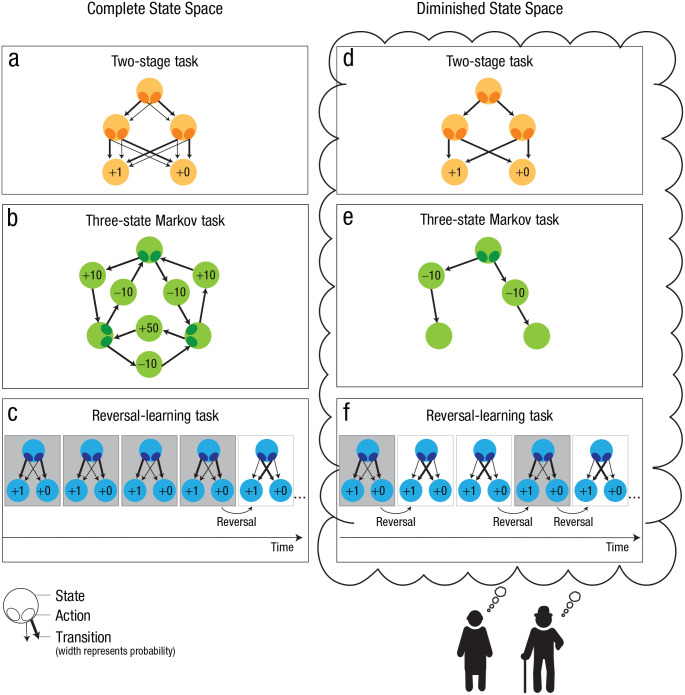
Complete state spaces of experimental tasks (a–c) and potential versions of diminished state spaces (d–f). The experimental tasks illustrated here are the two-stage task (a) as used in Eppinger, Walter, et al. (2013), the three-state Markov task (b) as used in Eppinger et al. (2015), and the reversal-learning task (c) applied by Hämmerer et al. (2019). The potential computational mechanisms resulting in the diminished state spaces illustrated in the figure are the pruning of unlikely transitions (d), reduced planning depth (e), and an inefficient updating of transition probabilities (f).

Empirical studies have used variants of so-called multistage Markovian learning tasks to study the interplay of model-free and model-based decision processes. A prominent version of such a paradigm is the *two-stage decision* task developed by Daw and colleagues (2011) (see Figure 2a). In this task, a decision at the first stage (State 1) determines a probabilistic transition to one of two states at the second stage. Participants must then decide between two actions and receive a reward. The participants are supposed to learn what the second-stage state with the highest reward is at a given time and how to transition from one state to the other to accumulate as many rewards as possible.

The choice data generated through this task can be used to estimate the degree of model-based behavior for each individual: that is, how well they integrate their representation of the state space with knowledge about the reward probabilities to accumulate as much reward as they can.

In younger adults, choice behavior in this task reflects a mixture of model-based and model-free decision processes, but there are substantial individual differences in the degree of model-based control (Daw et al., 2011). For example, it has been shown that individuals with higher working memory capacity engage in more model-based decision-making and are less susceptible to the detrimental effects of stress on this strategy (Eppinger, Walter, et al., 2013; Otto et al., 2013; Schad et al., 2014). Furthermore, in younger adults higher cognitive-control abilities are associated with greater model-based decision-making, indicating that the two behaviors may share a common set of underlying computational processes (Otto et al., 2015). Recent work also points to an association between the ability to infer latent structures in the environment and model-based control in the two-stage task (Markant, 2020; Rmus et al., 2019). Together, these results suggest that model-based control relies on the ability to represent and update an internal model of the task structure (the state space).

In contrast to younger adults, older adults rely predominantly on model-free learning strategies and show performance deficits under conditions that require model-based learning (Bolenz et al., 2019; Eppinger et al., 2015; Eppinger, Walter, et al., 2013; Ruel et al., 2022). Furthermore, findings from these studies suggest that the degree to which older adults shift toward model-free strategies depends on their ability to represent the state space of the task. Results by Bolenz et al. (2019) show evidence for an adjustment of decision strategies after changes in the task structure (the state space) in younger adults. Furthermore, these adjustments were associated with slowing of reaction time after changes in the task structure, indicating that the behavior reflects a deliberate adaptation to changes in the state space. In contrast, older adults show deficits in the updating of task representations as well as less slowing of reaction time after changes in the task structure, supporting the idea of deficits in state space representations. In a recent study, Ruel et al. (2022) investigated age differences in model-based decision-making and manipulated the probabilistic transition structure in two demand conditions. Consistent with the data by Bolenz et al. (2019), the behavioral results showed substantial age-related deficits in model-based decision-making under high demands on the representation of the task structure (60% common transitions, 40% rare transitions). Even under low representational demands (80% common, 20% rare transitions), older adults showed reduced model-based decision behavior when compared to younger adults.

Similar findings come from a study on age differences in reversal learning, which showed that older adults are less able to converge on stable task representations and that this deficit is independent of age differences in outcome processing (Hämmerer et al., 2018). It is important to note that although the sequential decision tasks applied in the studies outlined above are similar, in that they can be described as a Markov decision process, they also differ in many important respects: the experimental tasks used in Eppinger, Walter, et al. (2013) and Ruel et al. (2022) involved probabilistic transition structures. In contrast, in the study by Bolenz et al. (2019), deterministic transition structures were used. In this study, the transition structure was manipulated in two conditions—a stable-transition condition in which the transition structure remained constant across trials within a block, and a variable-transition condition in which the state transition mappings reversed every 6 to 14 trials within a block. The studies by Eppinger et al. (2015) and Wittkuhn et al. (2018) applied a task with deterministic transition structures across three (instead of two) states and with rewards as well as punishments. In the study by Bolenz et al. (2019), performance on the task (reflected in monetary payouts) depended on the degree to which participants engaged in model-based behavior, whereas in the studies by Eppinger, Walter, et al. (2013) and Ruel et al. (2022) performance was independent of the degree of model-based behavior. Thus, the state space theory is partly based on an empirical generalization, but the experimental designs of the underlying tasks vary substantially, which allows for a detailed examination of the deficits experienced by older adults.

With that being said, we do not want to imply that age-related differences in learning and decision-making are due only to a diminished representation of state spaces. For example, results of several studies indicate that older adults might have difficulties in building up reward-value representations, particularly under high degrees of reward uncertainty (Chowdhury et al., 2013; Eppinger et al., 2011; Eppinger, Schuck, et al., 2013; Lerner et al., 2018). Yet these deficits do not seem to be the primary sources of the shift from model-based to model-free control that is observed in healthy aging. In the study by Eppinger, Walter, et al. (2013), the random walks that determined the reward probabilities at the second stage of the task were manipulated. More differentiated reward probabilities (greater differences in the random walks that determined the rewards) did support model-based behavior in younger adults, but not older adults.

### Psychological mechanisms underlying diminished state space representations

A straightforward explanation for the observed age-related deficit in model-based learning might be the well-documented age differences in working memory capacity. Findings by Otto et al. (2013) show a significant positive association between working memory and model-based control in younger adults. Results from Eppinger, Walter, et al. (2013) replicate these findings in the young; however, they show no such correlation in older adults. Furthermore, when researchers controlled for differences in working memory capacity, age differences in model-based behavior remained statistically significant, suggesting that working memory capacity does not fully explain age differences in model-based behavior. Again, this is not to say that model-based control is independent of working memory, as the evidence for such a positive relationship between model-based behavior and working memory in young adults is compelling (Eppinger, Walter, et al., 2013; Otto et al., 2015). However, it seems that working memory may not be the primary reason for the limitations in model-based behavior observed in older adults.

An alternative (although not mutually exclusive) explanation for the shift from model-based to model-free behavior with age might be age-related deficits in the consolidation of state space information (Schuck & Niv, 2019). Using a sequential decision task in combination with multivariate pattern analyses of functional magnetic resonance imaging (fMRI) data, Schuck and Niv (2019) show evidence for a sequential replay of task states in the hippocampus. Furthermore, these replay mechanisms seem to drive the learning of complex state spaces in other brain areas, such as the OFC (see also Momennejad et al., 2018). Accumulating evidence suggests that replay also occurs in cortical areas and can be decoded from fMRI resting-state data (Wittkuhn & Schuck, 2021). Work by Liu et al. (2021) supports this view by showing a backward cortical replay signal in magnetoencephalography data that is associated with learning performance. They suggest that this replay signal may provide a mechanism for solving nonlocal credit assignment problems during model-based learning by connecting actions and outcomes across intervening states (Liu et al., 2021).

To summarize, it seems very likely that the replay of state sequences is critical for the construction and use of state space representations. There is indirect evidence from the aging and episodic-memory literature that shows a reduced association between neural reinstatement signals in the medial temporal lobe and memory performance in older adults (Stawarczyk et al., 2020). However, to our knowledge, the direct relationship between the replay signals described above and learning and memory abilities in older adults has yet to be examined. In the following two sections we will consider potential computational implementations of the diminished state space theory and consider how impoverished state space representations might be reflected in the brain.

### Computational mechanisms

In computational reinforcement-learning models, a state space can be represented by a set of states in the environment, a set of possible actions and the transition probabilities *P*(*s′|s,a*) that specify the subjective assumptions about how likely performing action *a* in state *s* will lead to the new state *s′* (Figs. 2a, 2b, and 2c). By means of these transition probabilities, the expected reward *Q*(*s,a*) for a state-action pair can be computed as the weighted mean of the reward in subsequent states. The reward of a subsequent state is composed of the reward immediately available in this state *r*(*s′*) and the temporally discounted expected future reward from choosing the most valuable action in this state:

$$Q\left(s,a\right)=\;\sum_{s\prime }P\left(s^{\prime }|s,a\right)[r\left(s^{\prime }\right)+\gamma \underset{a\prime }{\max}Q(s^{\prime },a^{\prime })].$$

Note the recursive nature of this computation: To evaluate the potential consequences of an action, one must consider the immediately subsequent states as well as all possible subsequent states after two, three, or more steps. Thus, computing a reward expectation is guided by the part of the state space representing the transition probabilities from the current state, and it also requires a representation of the transition probabilities for all states that can potentially be reached at some point in the future.

We propose that the diminished state space theory of human aging could be reflected in two different ways in computational models: first as an incomplete or inaccurate representation of states, actions, or transition probabilities, and second as an inefficient updating of these transition probabilities.

#### Simplified representations of the state space

Older adults might have difficulties with maintaining information about differences in transition probabilities (e.g., “this action will lead with 80% probability to state X and with 20% probability to state Y”). Therefore, rather than trying to accurately represent the state space, they may only maintain information about the states that are connected and consider all these connections to be equally likely (e.g., “this action will lead to either state X or state Y with equal probability”). The idea that older adults might simplify their representation of transition probabilities receives support from the study by Ruel et al. (2022), which showed that older adults represented transition probabilities as almost equally likely even when in fact one transition was five times more likely than the other possible transition. Alternatively, it could be that older adults simplify their representation of the state space by pruning branches of the decision tree with aversive outcomes or with low frequencies (Fig. 2d), thus reducing the number of states or actions. Similarly, older adults might restrict the planning depth of their state space (Fig. 2e), for example, by representing only information of immediately subsequent states but not of states thereafter (see Huys et al., 2012, 2015, and Keramati et al., 2016, for findings on these strategies in younger adult samples). Findings also show that humans can use *successor-representation learning*, a strategy that relies on stored predictions of future states (instead of transition probabilities between all possible states) and is therefore simpler than a model-based strategy while preserving a lot of its flexibility (Momennejad et al., 2017). Finally, recent work by Ho et al. (2022) suggests that people adaptively construct simplified representations of cognitive tasks when planning action sequences (Ho et al., 2022). The authors refer to these as *task construals*, and it is possible that older adults are less flexible in adjusting the complexity of these representations to task demands. Taken together, although there is considerable evidence for simplified representations of state spaces in older adults, the mechanisms that govern these simplifications are not clear yet and need to be established in future studies.

#### Inefficient updating of transition probabilities

One of the major benefits of model-based decision strategies is that they can flexibly adapt to changes in the task structure. However, in order to do so, these changes need to be incorporated by updating the transition probabilities. Previous computational accounts have modeled this with state prediction errors and transition learning rates similar to temporal-difference learning (Bolenz et al., 2019; Gläscher et al., 2010). The magnitude of the transition learning rate then determines the degree to which experienced transitions are disregarded as oddballs or random perturbations (learning rate = 0) or interpreted as indicators for changes in the underlying task structure (learning rate close to 1). There is no universal value for an optimal transition learning rate. Rather, the transition learning rate has to be flexibly adjusted on the basis of the statistical properties of the environment (D’Acremont & Bossaerts, 2016; Nassar et al., 2019). Age-related deficits in the flexible adjustment of transition learning rates might lead to an insufficient (i.e., rigid) representation of the state space (Fig. 2e), consistent with the predictions of the diminished state space theory. However, age differences in model-based behavior are also observed when transition probabilities are deterministic (Bolenz et al., 2019; Eppinger et al., 2015), which indicates that diminished state space representations are not due to deficits in learning probabilistic state transitions alone.

One reason for an insufficient updating of learning rates may be age-related alterations in episodic-memory replay that is necessary for keeping track of the changes in state transitions (Olafsdottir et al., 2018; Schuck & Niv, 2019). Previous work suggests a role of reward prediction errors in prioritizing replay of state transition sequences (Momennejad et al., 2018). However, whether state prediction errors have a similar effect in supporting replay is currently unclear. Another reason for reduced state transition learning rates in older adults could be their greater tendency to perseverate on previous predictions (Bruckner et al., 2021; Nassar et al., 2016). A greater rigidity of internal state spaces might be beneficial if shifts of representations lead to biases in judgments, such as in prevalence-induced concept change (Devine et al., 2022). However, it is maladaptive in environments in which performance depends on a flexible adjustment of state space representations. To summarize, each of these computational explanations seems consistent with what is seen behaviorally (Bolenz et al., 2019; Hämmerer et al., 2018; Ruel, Bolenz, et al., 2021). Yet the exact mechanism that leads to these deficits in updating is unclear.

We have presented two potential computational explanations for diminished state space representations in older adults: (a) simplified representations of state spaces and (b) an inefficient updating of state transition probabilities. It is important to note that these explanations are neither mutually exclusive nor completely interchangeable. That is, older adults might have a simplified representation of the task structure even when no updating of the transition probabilities is needed (e.g., the stable-transitions condition in Bolenz et al., 2019). Similarly, if older adults are simply slower at incorporating state prediction errors in their internal model (inefficient updating), they should nevertheless eventually converge on the same mental representation of the task as younger adults. One way to shed more light on the question about the computational mechanisms underlying diminished state space representations in older adults could be to examine their neurobiological correlates.

### Neurobiological mechanisms

Several recent studies tried to establish the neural processes underlying goal-directed decision-making. Results of this work suggest that the OFC (Nogueira et al., 2016; Schuck et al., 2016; Wilson et al., 2014) and the hippocampus (Gershman & Daw, 2017; Lengyel & Dayan, 2007; Schuck & Niv, 2019; Stachenfeld et al., 2017) play key roles in the representation and updating of state spaces during model-based decision-making. In what follows, we go over recent findings on the role of each of these areas in goal-directed behavior and discuss how aging might affect the neural mechanisms underlying model-based learning and decision-making.

#### Hippocampus

Seminal findings by O’Keefe and Nadel (1978) demonstrated that activity in the rat hippocampus reflects the use of cognitive maps during spatial navigation (O’Keefe & Nadel, 1978). More recent work by A. Johnson and Reddish (2007) showed that these hippocampal representations are not static but are updated as the animal moves along its path in the maze, suggesting that spatial navigation is an active process that is likely controlled by other cognitive mechanisms (A. Johnson & Reddish, 2007). Situated in the medial temporal lobe, the human hippocampus appears to play a similar role as the rat hippocampus by learning locations relative to a spatial boundary (Doeller et al., 2008). However, in humans the hippocampus has also been associated with more abstract (i.e., not exclusively spatial) representations of episodic information (Deuker et al., 2016; Shohamy & Wagner, 2008; Tavares et al., 2015). These findings led to the hypothesis that the hippocampus may play a role in representing state space information necessary for model-based reinforcement learning (Gershman & Daw, 2017; Lengyel & Dayan, 2007; Schuck & Niv, 2019). More specifically, the hippocampus has been suggested to be involved in the representation and planning of sequential actions necessary to achieve a certain goal. To gain a better understanding of how the brain learns these sequential state-action contingencies, Bornstein and Daw (2013) had participants view a series of images in which the next image to be presented depended probabilistically on the current image that they observed. At test, participants were asked to use the knowledge they had gained in the first phase of the task (regarding the sequential contingencies between stimuli) to guide their choice between two images in order to maximize reward (Bornstein & Daw, 2013). The neuroimaging analyses revealed that the hippocampus and nearby cortical areas are involved in the representation of state contingencies during model-based decision-making. Work by Kaplan and colleagues (2017) echoed these findings by demonstrating that in a spatial sequential choice task the hippocampus is part of a network that supports planning by maintaining representations of current and potential future choices (Kaplan et al., 2017). More recently, in a neuropsychological study, Vikbladh and colleagues (2019) found that the degree to which participants’ planning was impaired was related to the amount of damage to the right hippocampus (Vikbladh et al., 2019). Further, in line with findings in rodents, recent work has shown that sequential replay of experiences in the hippocampus and cortex during decision-making also occurs in humans (Liu et al., 2019, 2021). So it seems that the hippocampus may be involved in representing two important pieces of information necessary for model-based planning and decision-making: (a) the transition probabilities between different states and (b) the states that can be potentially reached in the future. What remains unclear from the current work on the involvement of the hippocampus in goal-directed planning and choice behavior is the degree to which it can be extended beyond the spatial domain.

#### OFC

Another brain region proposed to be involved in goal-directed decision-making is the OFC. The OFC is located on the ventral surface of the prefrontal cortex and has been previously suggested to play a key role in the representation of reward value, or more generally, in hedonic experience (Kringelbach, 2005; Rolls, 2000; Wallis, 2007). However, recent work points into a different direction and suggests that the OFC is involved in the representation of an agent’s current location within an abstract cognitive map of a task (Bradfield et al., 2015; Schoenbaum et al., 2009; Wilson et al., 2014). Recent empirical evidence provides support for this theory. For instance, Schuck et al. (2016), using multivariate pattern analysis, found patterns of activity in the OFC during a 16-state decision-making task that contained unobservable information about the participants’ location in a cognitive map of the task. Performance on the task depended on memory of past events and knowledge about the current phase of the task. In line with previous animal studies (Takahashi et al., 2011), the authors suggested that the human OFC may represent information about locations in a mental (i.e., nonspatial) map of a task that is composed of hidden or partially observable states. More recently, S. C. Y. Chan et al. (2021) tested this prediction empirically. Using univariate and multivariate activity fMRI analyses, they demonstrated that activity in the OFC correlates with experienced state transitions. Their findings reveal that the OFC is also involved in learning a state-to-state transition structure, necessary for accurate planning during model-based decision-making (S. C. Y. Chan et al., 2021).

What about the interplay of the OFC and hippocampus during decision-making? Several findings indicate that the hippocampus and the OFC support separate functions, such as episodic memory (Burgess et al., 2002; Tulving & Markowitsch, 1998) and the representation of subjective (reward) value (Peters & Büchel, 2010). However, there is also evidence that points to complementary functions when it comes to decision-making. Work by Wang et al. (2020) indicates that the OFC and hippocampus represent partially overlapping information: The hippocampus may be involved in the initial acquisition of information for the creation of a cognitive map, whereas the OFC seems to maintain these cognitive maps and to guide behavior (Wang et al., 2020; see also Schuck & Niv, 2019; Wimmer & Büchel, 2019). Further, work by E. L. Johnson et al. (2022) suggests that the OFC, similar to the hippocampus, supports an understanding of temporal-order information (E. L. Johnson et al., 2022). In line with previous work by Barbey et al. (2011), these findings highlight the role of the OFC in working memory and temporal cognition (Barbey et al., 2011). If we return to the train ticket example, the hippocampus appears to help users represent the overall relationship between states, but the OFC may play a role in helping them keep track of the states they are currently in (i.e., the menu) on the basis of previous actions (i.e., choices) and previously visited states.

Taken together, current findings indicate a role for the OFC as well as the hippocampus in creating, maintaining, and updating nonspatial state space representations during goal-directed decision-making. Specifically, these findings suggest that the OFC and hippocampus represent partially overlapping information and that interactions between these two regions support model-based planning and decision-making. Although the hippocampus has been suggested to be involved in the creation of a cognitive map of the state space and the transitions between them, the OFC appears to help the agent use this map to guide behavior.

### The effects of aging on hippocampal and OFC function

A considerable body of structural magnetic resonance imaging work indicates that prefrontal regions and the hippocampus deteriorate as we age (Hedden & Gabrieli, 2004; Raz et al., 2005; West, 1996). In line with the structural findings, fMRI research on episodic and working memory suggests an age-related underrecruitment of various areas in the prefrontal cortex, including the OFC (Rajah & d’Esposito, 2005; Resnick et al., 2007). This underrecruitment of the prefrontal cortex, and particularly the OFC, could limit top-down activation of the model-based learning network, leading older adults to rely more on simpler, model-free decision strategies. According to the neurobiological evidence, a shift away from model-based learning could be related to changes in the function of (a) the hippocampus, because of its involvement in the representation of state spaces, and (b) the OFC, in line with its role in helping the agent use and update these representations.

Research on age-related changes in memory has long shown that healthy aging is associated with a decrease of contextual (episodic) memory, whereas memory for content (semantic information) remains more intact (see Spencer & Raz, 1995). To our knowledge, the relationship between age-related changes in hippocampal structure and function and model-based behavior has not yet been established. Nevertheless, in view of the findings in younger adults, there is concern that hippocampal decline may lead to difficulties representing state transitions required for a complete cognitive map of the state space (Bornstein & Daw, 2013). Findings by Liu et al. (2019, 2021) suggest that hippocampal and cortical replay is involved in the creation of a mental model of the decision-making environment and by this may support model-based learning. Age-related decline of the hippocampus and the associated replay mechanisms may contribute to deficits in goal-directed decision-making during aging. The neuropsychological findings of Vikbladh et al. (2019) suggest that the degree of hippocampal deterioration would affect both decision-making and spatial navigation.

Beyond the effects on the hippocampus, healthy aging also strongly affects the structure and function of prefrontal regions (West, 1996). Specifically, with increasing age, various prefrontal regions appear to be underrecruited, which may lead to difficulties in older adults in using and updating their state space representation. Findings from an fMRI study by Eppinger et al. (2015) support this view, showing that impairments in older adults in learning to predict future rewards are associated with prefrontal deficits in extracting sequential state transition structures (Eppinger et al., 2015; Wittkuhn et al., 2018). A study by Wittkuhn et al. (2018) replicated the behavioral results in older adults and showed that the age-related decline in the learning of state transition structures can be mimicked in younger adults by inhibiting prefrontal cortex function using repetitive transcranial magnetic stimulation (rTMS; Wittkuhn et al., 2018). Moreover, recent findings indicate that even when demands on the representation of the state transition structure are reduced, older adults show marked difficulties learning probabilistic transitions between states, compared to younger adults. Consistent with the behavioral deficits, electroencephalography (EEG) data showed blunted trial-by-trial neural responses following unexpected state transitions in older compared to younger adults, whereas neural responses to reward feedback seemed intact in older adults (Ruel et al., 2022). These results indicate that the older adults fail to realize changes in the task structure (as indicated by the lack of state prediction errors in the ERP), whereas they seem to be able to adjust behavior as a function of reward feedback.

Taken together, the current findings suggest that the observed age-related deficits in older adults in the representation and updating of state transition structures (state spaces) result from functional decline in the OFC and possibly the hippocampus. Although this neurobiological explanation is consistent with behavioral findings and computational accounts of decision-making in older adults, there is a strong need for further research to support this hypothesis. We suggest that future work should first examine the OFC and hippocampus independently in order to improve our understanding of the impact of aging on these regions and the effects this has on older adults’ ability to engage in goal-directed learning. On the basis of the findings in younger adults, we predict that the hippocampus may be involved in helping the user represent transitions between states, whereas the OFC may play a role in helping the user apply and update state space representations. Methods such as multivariate pattern analyses and computational modeling may help develop our mechanistic understanding of the representational capacities of the hippocampus and OFC.

### Summary of the theory

In the diminished state space theory of human aging, we propose that age-related limitations in complex goal-directed behavior may result from an underlying deficit in the representation of state spaces of cognitive tasks (see Fig. 1). We provide evidence from behavioral and cognitive neuroscience studies that support our theory, and we make first steps toward potential computational implementations of such deficits. As outlined in the introduction, in industrialized high-tech societies there is an increased need for complex learning and decision-making abilities. However, these abilities clearly decline with advancing age, and the societal impact of this decline is magnified given the rising numbers of older adults in most developed countries. To be able to counteract the implications of these age-related limitations, we need to understand the underlying cognitive, computational, and neurobiological mechanisms. Here we propose that diminished state space representations might be one source of these deficits.

The diminished state space theory could be understood as an intermediate-level theory that provides an interface between lower-level deficits in basic cognitive processes and interactions with the environment. We think that this is the strength of the theory, rather than a weakness. In the last decades, several theories have been proposed to explain cognitive aging in terms of cognitive primitives, such as working memory (e.g., Kirasic et al., 1996; D. C. Park et al., 2003), inhibition (Lustig et al., 2007), or episodic memory (e.g., Shing et al., 2010). However, these processes (and age-related changes therein) tend to be positively correlated with each other. This suggests that there might be a shared common factor behind all the observed age differences in cognitive abilities (Salthouse, 1988; Verhaeghen & Salthouse, 1997). The problem with the process-specific aging theories is that they may not generalize, and they may be difficult to test in a rigorous manner across different cognitive domains. The issue with common-factor theories of cognitive aging seems to be that that one factor has not yet been identified, and it is unclear how such a common cognitive factor might be reflected on the computational and neurobiological level. Instead of engaging in this debate, we propose an intermediate-level theory in which multiple causes (including, but not limited to, deficits in working memory, cognitive control, or episodic memory) might contribute to a more global deficit in the representation of state spaces of cognitive tasks. It is currently unclear whether there is a unique effect of aging on state space representations or whether diminished state spaces reflect the joint contribution of age differences in the cognitive primitives mentioned above. To address this question it requires three (interrelated) methodological approaches: (a) multivariate longitudinal studies that include a broader range of control variables (in particular, cognitive-control abilities and episodic memory); (b) age-comparative experimental manipulations of state space representations, such as manipulating the size of the state space or the need for an updating of the state space, across a range of cognitive abilities—learning, cognitive control, and episodic memory; and (c) representational similarity or multivoxel pattern analyses of EEG or fMRI data. These analyses will uncover age differences in the neurobiological correlates of state space representations and how they relate to age differences in cognitive abilities.

### Specific predictions of the theory, generalization, and pathways for application

#### Specific predictions

In the paragraph on computational mechanisms, we suggest that diminished state space representations in older adults may result from the pruning of branches of the decision tree, from the use of simplified task construals, or from the application of heuristic strategies such as successor representation learning. The pruning of branches of the decision tree is difficult to assess directly in experimental paradigms but could be studied indirectly by testing memory for state information encoded during learning. Differences in subjective task representations (construals) have been investigated in a recent study by Ho et al. (2022) using a goal-directed maze-navigation paradigm. Using these types of tasks in older adults should reveal that deficits in forward planning and navigation in elderly people result from oversimplified task construals. Finally, recent work has studied the use of successor representation learning as a cognitively less demanding alternative decision strategy (Momennejad et al., 2017). This approach could be used in older adults in combination with neural measures of off-line replay (Momennejad et al., 2018). The hypothesis would be that diminished state spaces in older adults reflect the use of successor representation learning and are associated with differences in replay. In the paragraph on the computational mechanisms underlying deficits in state space representations, we also suggest that an inefficient updating of transition probabilities may be one source of impoverished state space representations in older adults. This could be tested empirically by comparing neural (EEG or magnetoencephalography) correlates of state prediction errors of different magnitude in conditions in which such errors can or cannot be used for learning. This would allow researchers to dissociate age-related deficits in state-prediction-error signaling itself from an inappropriate updating of state predictions in older adults (see Nassar et al., 2019).

#### Generalization to other cognitive domains

One cognitive domain in which older adults show consistent deficits is cognitive control. An important aspect of cognitive control in the Stroop task or the task-switching paradigm is to be able to accurately represent the task sets (defined as all stimulus–action mappings required to perform a task) and to keep the representations of task sets as separate from each other as possible (see Musslick & Cohen, 2021). The diminished state space theory suggests that task-set representations diminish with aging. This should lead to specific impairments in task switching in older adults: On the basis of the theory, we predict that a diminished representation of task sets should affect performance on switch and repeat trials during task switching (general or global switch costs) in older adults, leaving the actual process of switching unaffected (specific or local switch costs). This is consistent with what is observed empirically in the aging and task-switching literature (for a meta-analysis, see Wasylyshyn et al., 2011). Future studies could try to test the predictions of the diminished state space theory in task-switching paradigms by increasing the load on the representation of the state space (e.g., by increasing the number of tasks) or by manipulating the local transition probabilities of the task sets.

The diminished state space theory could also be applied to explain age-related deficits in reversal learning or performance on tasks such as the Wisconsin Card Sort Test. Work by Hämmerer et al. (2019) has suggested that older adults tend to overestimate the likelihood of reversals during learning. They interpret their findings as an age-related deficit in the ability to build up accurate representations of optimal choice behavior (Hämmerer et al., 2019). Viewed through the lens of the diminished state space theory, the underlying deficit could be explained by difficulties in the establishment of differentiated state representations. Recent data in rats suggest that state representations during reversal learning can be decoded from activity of orbitofrontal neurons (Bartolo & Averbeck, 2020; Stalnaker et al., 2021). In humans, the work by Schuck and Niv (2019) showed similar representations in the OFC, which were associated with task performance in a variant of a reversal-learning task. In view of the work by Schuck and Niv (2019), the most straightforward way to test the diminished state space theory in task-switching and reversal-learning tasks would be to try to decode task sets and attentional states from fMRI or EEG data during performance of these tasks. Accordingly, these state representations should be less differentiated in older adults, and the representational deficits should predict learning and task-switching performance in the elderly.

#### Applications

To exemplify potential applications of the theory, we come back to the initial example of an older adult trying to purchase a train ticket. Apart from the in-person ticket counter, there are two ways of purchasing a train ticket: at a ticket-vending machine, or using a computer app. Both options are characterized by complex state spaces that are not directly observable and have been shown to be a major challenge for older adults (Schreder et al., 2012; Sengpiel, 2016). One important feature that distinguishes vending machines from apps is the degree to which the device can learn about the preferences and cognitive abilities of their users; this in turn determines design recommendations. One obvious way to support model-based decision-making in older adults using these devices would be to reduce overall representational load by simplifying the state space (the number of available states and actions). Another way could be to foreshadow transitions to subsequent states (menus) or to provide an a priori map of the state space: That way one could offload the computational cost of having to internally represent and update state transitions. Lindenberger and Mayr (2014) suggested that environmental cues (as on a menu of a ticket-vending machine) should be compatible (functionally related to the goal of the agent) and distinctive (they should not coactivate competing actions). Compatibility in multistage decision-making would mean that in the beginning of the purchasing process state decision trajectories that are in line with the predicted preferences of the agent would be made more available. Furthermore, the state space could be adapted so that it matches the task construals or the simplifying decision strategies of the (aged) user. Distinctiveness means that state transition structures need to be unambiguous and predictable. However, as outlined above, surprising outcomes (state transitions or rewards) may trigger learning. Thus, it could be useful to consider ways to induce surprising transitions (e.g., to rewarding end states) to support learning in the agent.

The potential applications of the diminished state space theory outlined above center on reducing load on state space representations in older adults. However, there are two interrelated trade-offs to be considered: First, in order to maximize learning in older adults (and other age groups), it may be important to account for individual cost-benefit evaluations and to adjust the decision environment accordingly (see Devine et al., 2021; Ruel, Devine, & Eppinger, 2021). Second, an overreliance on environmental support (such as the adaptive algorithms outlined above) may diminish cognitive resources in older adults (see Lindenberger & Mayr, 2014). Finally, there are ethical and data-security concerns that need to be considered. After all, the interest of the transportation company may not align with that of the customer, and the algorithms underlying such technology should remain transparent to its users.

### Relationship to other theories

The diminished state space theory ties in with suggestions that older adults rely more on external information across several psychological domains. Lindenberger and Mayr (2014) proposed that this greater reliance on environmental support might be a (mal)adaptive adjustment to deficits in the ability to trigger and maintain cognitive representations (Lindenberger & Mayr, 2014). Here we provide a first explanation for what these representations are and how they might be implemented computationally and on a neurobiological level. Future work should try to capture differences in the distinctiveness of state space representations between age groups (e.g., through multivariate pattern analyses) and use this information to predict shifts in decision strategies as well as a greater reliance on environmental control in older adults. The current theory can be seen as being foreshadowed by past work on working with memory (Moscovitch, 1994) in which the author suggested that memory performance does not reflect the operation of a single system but depends on the interaction of processes within a network of interrelated components, including sensory areas, basal ganglia, the hippocampus, and the prefrontal cortex. The diminished state space model also resonates with the neuronal gain control theory of human aging, which proposes that less differentiated cognitive representations in older adults may result from deficient dopaminergic input to cortical areas (Li et al., 2001). However, so far there is no direct evidence for an association between age-related decline in the dopamine system and deficits in state space representations in the OFC and hippocampus. This should be established in the future. Our theory also seems compatible with the proposal that age-related changes in the locus coeruleus norepinephrine system may underlie cognitive decline in older adults (Mather & Harley, 2016). Mather and colleagues suggest that the phasic norepinephrine signals from the locus coeruleus (LC) may shift cognitive representations in accordance with arousal levels (Mather et al., 2018). Specifically, norepinephrine may modulate cortical excitation and inhibition, which in turn guides selective processing of information during learning. Therefore, age-related degeneration of the LC system may result in impairments in selective attention and downstream effects on model-based learning. Although some research shows that older adults have a less reliable attentional filter than other age groups (Dahl et al., 2020), future research should consider this research question using indicators of LC function (such as pupillometry and EEG signals) as well as structural MRI measures of LC integrity to predict age-related changes in state space representations. Finally, we would like to conjecture that deficits in state space representations may not necessarily imply suboptimal learning and decision behavior in older adults. From a resource-rational perspective (Lieder & Griffiths, 2020), it could well be that shifts in behavioral strategies with age reflect an optimal adaptation to internal constraints (diminished state space representations) in older adults (Devine et al., 2021; Ruel, Devine, & Eppinger, 2021). Finally, it seems plausible that age-related changes in the selectivity of neural processing (neural dedifferentiation; Koen et al., 2020; Koen & Rugg, 2019) might be one of the neurobiological mechanisms underlying diminished state space representations in older adults. However, there are also important differences between the neural dedifferentiation theory and the diminished state space theory that need to be considered: First, the complexity of the state spaces that are under study is very different. The neural dedifferentiation theory is primarily based on evidence from studies on face and object perception (Burianová et al., 2013; Goh et al., 2010; J. Park et al., 2012). Even in work that focuses on more complex cognitive functions (such as episodic memory), the corresponding tasks consist of very small state spaces (in most cases, one state and two possible actions; Goh et al., 2010; Koen et al., 2019; Pauley et al., 2023). The state spaces and cognitive tasks that we are referring to in the diminished state space theory are far more complex and consist of multiple interdependent states, actions, and outcomes (see Fig. 2). One of the central predictions of the diminished state space theory is that behavioral deficits in older adults should increase with the size of the state space that needs to be represented. This effect should be much less pronounced in younger adults. In contrast, most of the literature on neural dedifferentiation suggests an age-invariant relationship between dedifferentiation and cognitive abilities (Koen et al., 2020). Second, in the diminished state space theory we focus on scenarios in which decisions at one state have implications for the subsequent states that individuals encounter, as well as the outcomes that are obtained in the future. In contrast, the empirical research that serves as a foundation for the neural dedifferentiation theory focuses on situations in which subjects make judgments on individual stimuli. In most cases these judgments are not associated with feedback or reward and, most importantly, they do not have implications for what is happening in the future. Finally, the neural dedifferentiation theory focuses on the link between age differences in neural dedifferentiation in functionally specific brain networks and age-related deficits in cognition (Koen & Rugg, 2019). The diminished state space theory does not assume that state space representations are implemented in activity in a single neural network with a specific function. Rather, the theory suggests that these representations are reflected in activity dynamics in configurations of multiple interrelated functional neural networks (M. Y. Chan et al., 2017; J. S. X. Chong et al., 2019). To summarize, we think that neural dedifferentiation might be one of neurobiological mechanisms underlying diminished state representations. However, at this point there are important differences between the theories in terms of the complexity of the psychological processes that are targeted, the static versus dynamic nature of the representations under study, and the fact that neural state space representations likely involve multiple interrelated neural networks rather than one functionally specific network. The exact relationship between the two theories is an open question. We think that the combination of methodological approaches that try to capture neural dedifferentiation (such as representational similarity analyses) as well as an exploration of the computational and experimental approaches that we put forward in the diminished state space theory may move us closer to an answer to this question.

## Conclusion

In industrialized high-tech societies there is an increased need for complex learning and decision-making abilities. However, these abilities decline with advancing age, and the societal impact of this decline is magnified given the rising numbers of older adults in most developed countries. Relying on findings from age-comparative computational- and cognitive-neuroscience studies, we propose that age-related impairments in complex goal-directed behavior result from deficits in the representation of state spaces of cognitive tasks in the OFC and hippocampus.

## Transparency

*Action Editor:* Nora S. Newcombe

*Editor:* Interim Editorial Panel
